# Pleomorphic Adenoma: Extracapsular Dissection vs. Superficial Parotidectomy—An Updated Systematic Review and Meta-Analysis

**DOI:** 10.3390/medsci13030104

**Published:** 2025-07-31

**Authors:** Giovanni Salzano, Veronica Scocca, Stefania Troise, Vincenzo Abbate, Paola Bonavolontà, Luigi Angelo Vaira, Umberto Committeri, Jerome R. Lechien, Sara Tramontano, Vitanna Canterino, Giovanni Dell’Aversana Orabona

**Affiliations:** 1 Maxillofacial Surgery Unit, Department of Neurosciences, Reproductive and Odontostomatological Sciences, University Federico II of Naples, Via Pansini 5, 80131 Naples, Italy; giovannisalzanomd@gmail.com (G.S.); stefania.troise@unina.it (S.T.); giovanni.dellaversanaorabona@unina.it (G.D.O.); 2 Maxillofacial Surgery Unit, Department of Clinical and Surgical Medicine, University Federico II of Naples, Via Pansini 5, 80131 Naples, Italy; 3 Maxillofacial Surgery Operative Unit, Department of Medical, Surgical and Experimental Sciences, University of Sassari, 07041 Sassari, Italy; 4 Maxillofacial Surgery Unit, Santa Maria Hospital, 05100 Terni, Italy; 5 Department of Surgery, University of Mons, 7000 Mons, Belgium

**Keywords:** pleomorphic adenoma, superficial parotidectomy, extracapsular dissection, parotid gland surgery, benign tumor of the parotid gland

## Abstract

**Background/Objectives**: The aim of our study was to evaluate clinical outcomes in patients with small pleomorphic adenoma (PA) after extracapsular dissection (ED) versus superficial parotidectomy (SP). **Methods**: Following the PRISMA guidelines, a systematic review covering the years from 1950 to 2025 was conducted using the Pubmed/MEDLINE, Cochrane Library, Scopus, Ovid MEDLINE and Embase databases. A single-arm meta-analysis was performed to evaluate intraoperative capsular rupture, recurrence, transient and permanent facial nerve palsy, Frey’s syndrome, salivary fistula, seroma and hematoma of patients who underwent ED vs. those who underwent SP, and funnel plots were constructed to evaluate the robustness of the findings. **Results**: Of the 1793 identified papers, 21 articles met the inclusion criteria. The meta-analysis (2507 patients) reported the following: (1) the risk of recurrence is similar in patients treated with ED and SP; (2) the transient facial nerve palsy rate is lower after ED (*p* < 0.05), while the permanent facial nerve palsy rate is similar with ED and SP; (3) post-operative complications, especially Frey’s syndrome (*p* < 0.05), are more common after SP. **Conclusions**: Given the similar recurrence rate and the lower morbidity compared to SP, ED could be considered the treatment of choice for pleomorphic adenomas of the parotid gland that are up to 3 cm in size, mobile and located in the superficial lobe of the parotid gland.

## 1. Introduction

Salivary gland tumors are relatively rare, accounting for approximately 2% to 4% of all head and neck neoplasms [1]. The majority (75%) are benign, with the parotid gland being the most common site of origin (70%). Among these, pleomorphic adenomas (PAs) are the most common benign tumors of the salivary glands, accounting for 85% of all salivary gland neoplasms and 60% of benign tumors in the parotid gland [2,3]. The surgical management for parotid gland neoplasms has evolved over time due to the close relationship between the gland and the facial nerve, along with the high recurrence rate [4,5,6,7]. Initially, in the first half of the 20th century, enucleation was the primary approach. However, this technique often leaves behind remnants of the tumor or its (pseudo)capsule, leading to a high recurrence rate [8,9]. Currently, the gold standard treatment is total parotidectomy (TP) or superficial parotidectomy (SP), which has significantly reduced the recurrence rate [10,11]. However, these procedures involve the removal of a substantial portion of healthy parotid tissue and require facial nerve dissection, which can result in facial nerve injury and loss of parotid function [12]. To mitigate these risks, surgeons have increasingly adopted less invasive techniques like extracapsular dissection (ED) [1,13]. This approach focuses on excising only the tumor with a small margin of normal parotid tissue while preserving parotid function, thereby minimizing the risk of facial nerve injury and Frey’s syndrome [12,14,15] with an acceptable recurrence rate [1,12]. Other typical post-operative complications related to parotid gland surgery include defective wound healing, wound infections, dehiscence, hypertrophic scars, seromas, hematomas, sialocoeles, salivary fistulas, anesthesia and paresthesia around dermal incisions or the area supplied by the great auricular nerve, and gustatory sweating [1].

Surgery of the parotid gland remains a complex procedure due to the lack of consensus on the optimal treatment approach. However, the two most commonly employed techniques are ED and SP. The aim of this systematic review and meta-analysis is to compare the two surgical approaches, ED vs. SP, for the treatment of PA in terms of success, recurrence rate and post-operative complications.

## 2. Materials and Methods

The study followed the Preferred Reporting Items for Systematic Reviews and Meta-Analyses (PRISMA) guidelines. Since it involved a review of previously published studies, neither ethics approval nor informed consent was required. Furthermore, no review protocol was registered for this study. A population, intervention, comparison, outcome, studies (PICOS) statement was utilized to identify studies that met inclusion or exclusion criteria.

### 2.1. Search Strategy

The research covered the years 1950–2025 and included the Pubmed/MEDLINE, Cochrane Library, Scopus, Ovid MEDLINE and EMBASE databases. Relevant keywords, phrases and MeSH terms were tailored to meet the specific requirements of each individual database. An example of the search strategy is the one used for Pubmed/MEDLINE:

(“pleomorphic adenoma”[MeSH Terms] OR “pleomorphic adenoma”[All Fields] OR “benign mixed tumor”[All Fields]) AND (“extracapsular dissection”[All Fields] OR “superficial parotidectomy”[All Fields] OR “parotid surgery”[All Fields]) AND (“parotid gland”[MeSH Terms] OR “parotid gland”[All Fields] OR “salivary gland”[All Fields]). Then, a cross-reference search of the selected articles was conducted using the snowballing method to ensure the retrieval of all possible studies.

The last search was conducted on 20 March 2025.

### 2.2. Inclusion Criteria

#### 2.2.1. Participants

The patient population included male and female adults (aged 18 years or older) with pathologic diagnosis of pleomorphic adenoma. Selection criteria for pleomorphic adenoma were primary tumor, superficial lobe, size: less than 4 cm, unifocal, unilateral and capsular integrity. Studies that had a mixed cohort were only included if they had subset data on the target population.

#### 2.2.2. Intervention

The review considered studies where superficial parotidectomy or extracapsular dissection was performed. Following the Snow classification of 2000 [16], SP was considered as the removal of superficial parotid lobe with complete nerve dissection, and ED as the selective resection of the tumor with safe margins and complete nerve dissection.

#### 2.2.3. Comparison

The review compared superficial parotidectomy and extracapsular dissection. It included all studies that met the intervention and population criteria, regardless of whether a comparison between the two surgical techniques was made within the same study.

#### 2.2.4. Outcomes

The included studies had to report at least one of the following outcomes: intraoperative capsular rupture, recurrence rate and post-operative complications. Post-operative complications included were transient and permanent facial nerve palsy, Frey’s syndrome, salivary fistula, seroma and hematoma.

#### 2.2.5. Study Design

The review encompassed both experimental and quasi-experimental study designs, such as randomized controlled trials (RCTs) and non-randomized controlled trials. It also included both analytical and descriptive observational studies, including prospective and retrospective cohort studies, case–control studies, cross-sectional studies, and case series. Case reports were excluded from the systematic review.

### 2.3. Exclusion Criteria

Studies were excluded if they were not in English; were not available in full-text form; the article type was a case report, conference abstract, letter to the editor or book chapter; or they considered malignant tumors.

After the selection criteria were defined, 641 articles were screened, and 21 articles met the inclusion criteria.

### 2.4. Data Collection Process

The search was conducted independently by two investigators (V.S. and G.S.). References from the identified databases were merged, and duplicates were removed using the reference management software EndNote^®^ 21 (version 21.5). Articles were screened for relevance based on title and abstract, with those deemed appropriate being selected for full-text review. Any discrepancies between the screening authors were resolved through discussion until consensus was reached.

Systematic data extraction from the included studies was performed using a structured form, with data archived in a customized Excel^®^ (Microsoft Corp, Seattle, WA, USA) spreadsheet. One author (V.S.) independently compiled a standardized form to extract the following characteristics of the included studies: authors, year of publication, study design, number of patients with pathological diagnosis of PA, mean age, mean follow-up time, mean tumor size, number of EDs and/or SPs, capsular rupture, recurrence, transient and permanent facial nerve palsy, Frey’s syndrome, salivary fistula, seroma and hematoma. The accuracy of the extracted data was verified by another author (G.S.).

### 2.5. Data Synthesis and Analysis

All articles included in the qualitative analysis were then included in the meta-analysis.

Clinical measures were reported as provided by the individual studies. When the mean follow-up time was not available, the median measure was used.

A single-arm meta-analysis was performed for capsular rupture, recurrence, transient and permanent facial nerve palsy, Frey’s syndrome, salivary fistula, seroma and hematoma for both ED and SP groups. The results were presented as pooled estimates with 95% CIs, and a forest plot was generated for each outcome. Comparative analyses between the two groups were conducted using *p*-values to assess statistical significance across outcomes.

Cochran’s Q test was applied to assess heterogeneity between studies, and I^2^ was calculated as a measure of heterogeneity. The I^2^ value represents the percentage of total variation across studies caused by heterogeneity rather than by chance. According to the Cochrane criteria, values from 0% to 40% may signify low heterogeneity, 30% to 60% may represent moderate heterogeneity, 50% to 90% may represent substantial heterogeneity, and 75% to 100% may represent considerable heterogeneity.

A fixed-effect model was used if all studies came from a common population and if the effect size was not significantly different among the different trials. If the heterogeneity test produced a low probability value (Q-statistic, *p* < 0.05), then a more conservative random-effect model was used.

Publication bias was evaluated by visually inspecting the funnel plot, and the Egger’s linear regression test was used to statistically examine the asymmetry of the funnel plot.

All the analyses were performed using the R software for statistical computing (R version 4.4.2; “meta” and “dmetar” packages). Statistical significance was defined as *p* < 0.05.

### 2.6. Risk of Bias and Study Quality Assessment

Two authors (V.S. and G.S.) assessed the quality of each study using the Newcastle–Ottawa Quality Assessment Scale. To evaluate potential publication bias, a funnel plot was generated based on the effect size of each outcome. The overall quality of evidence for each primary outcome was assessed using the GRADE (Grading of Recommendations Assessment, Development and Evaluation) approach (see Table S1).

## 3. Results

### 3.1. Studies and Settings

**Figure 1:**
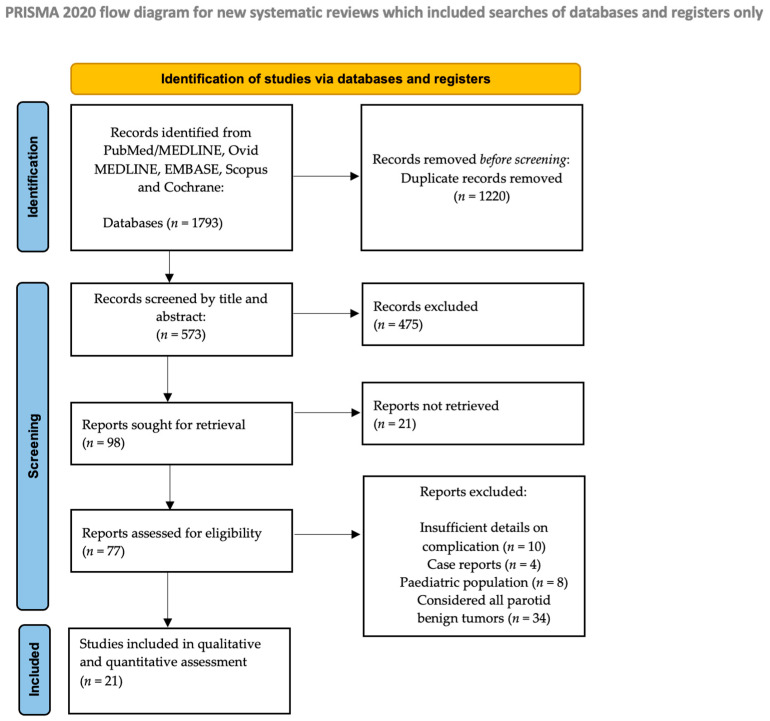
PRISMA flow diagram.

The study selection process is summarized in Figure 1. The data collection resulted in 1793 entries. Of 1793 articles, 122 were excluded before screening because they were duplicates. After the initial screening of the titles and abstracts, 475 articles were excluded because they were off-topic, and 21 because the full text was missing, while the remaining 76 were included for full text assessment. A total of 21 publications were included in the qualitative and quantitative (meta-analysis) synthesis.

### 3.2. Description of the Studies

The general characteristics of the studies are shown in Table 1. All the studies were in English, and all were retrospective except for four that were prospective [17,18,19,20]. Four studies were published in the 1990s [21,22,23,24], three in the 2000s [13,25,26], ten in the 2010s [18,19,20,27,28,29,30,31,32,33] and four in the 2020s [17,34,35,36].

### 3.3. Study Results

**Figure 2:**
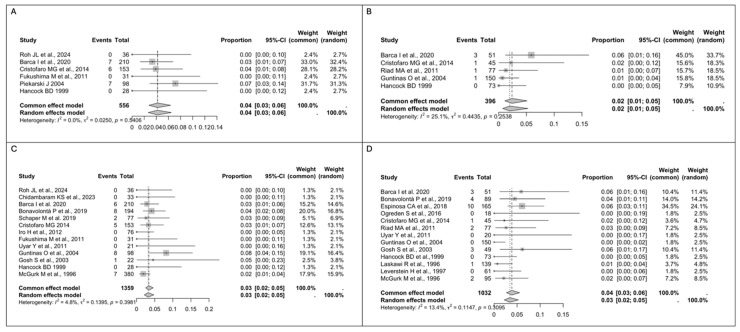
(**A**) Forest plot for intraoperative capsular rupture in the ED group. (**B**) Forest plot for intraoperative capsular rupture in the SP group. (**C**) Forest plot for recurrence in the ED group. (**D**) Forest plot for recurrence in the SP group. Abbreviations: CI, confidence interval. Data derived from Roh JL et al., 2024 [17], Chidambaram KS et al., 2023 [35], Barca I et al., 2020 [34], Qin H et al., 2020 [36], Bonavolontà P et al., 2019 [33], Schaper M et al., 2019 [32], Espinosa CA et al., 2018 [31], Infante-Cossio P et al., 2018 [18], Ogreden S et al., 2016 [30], Cristofaro MG et al., 2014 [29], Iro H et al., 2012 [28], Fukushima M et al., 2011 [27], Riad MA et al., 2011 [20], Uyar Y et al., 2011 [19], Guntinas O et al., 2004 [13], Piekarski J et al., 2004 [26], Gosh S et al., 2003 [25], Hancock BD et al., 1999 [24], Laskawi R et al., 1996 [23], Leverstein H et al., 1997 [22], McGurk M et al., 1996 [21].

A total of 2507 patients with pathological diagnosis of AP were included in the quantitative analyses. In total, 1359 patients underwent ED, while 1148 patients received SP. The rate of pooled intraoperative capsular rupture measured using a fixed-effect model was 4% in the ED group (*n* = 20/556, 95% CI 3–6), with a low between-study heterogeneity (I^2^ = 0%, Q = 0.0250, *p* = 0.5406) (Figure 2A), while in the SP group, the pooled rate reported using a fixed-effect model was 2% (*n* = 6/396, 95% CI 3–6), with a low between-study heterogeneity (I^2^ = 25.1%, Q = 0.4435, *p* = 0.2538) (Figure 2B). No statistically significant difference (*p* = 0.052) was observed between the two groups. For recurrence, the pooled analysis using a fixed-effect model revealed a rate of 3% in the ED group (*n* = 37/1359, 95% CI 2–9), with low between-study heterogeneity (I^2^ = 4.8%, Q = 0.1395, *p* = 0.3981) (Figure 2C). Similarly, in the SP group, the pooled rate reported using a fixed-effect model was 4% (*n* = 26/1032, 95% CI 3–6), with a low between-study heterogeneity (I^2^ = 13.4%, Q = 0.1147, *p* = 0.3095) (Figure 2D).

**Figure 3:**
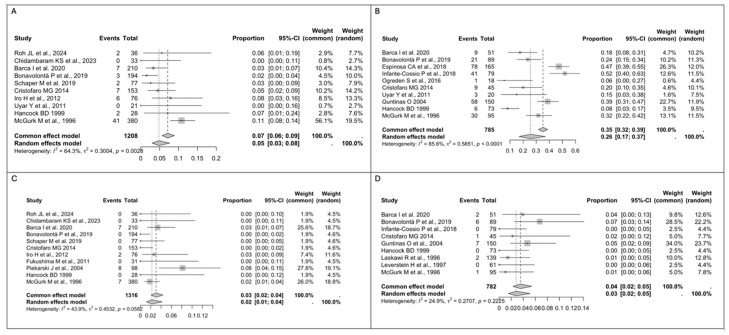
(**A**) Forest plot for transient facial nerve palsy in the ED group. (**B**) Forest plot for transient facial nerve palsy in the SP group. (**C**) Forest plot for permanent facial nerve palsy in the ED group. (**D**) Forest plot for permanent facial nerve palsy in the SP group. Abbreviations: CI, confidence interval. Data derived from Roh JL et al., 2024 [17], Chidambaram KS et al., 2023 [35], Barca I et al., 2020 [34], Qin H et al., 2020 [36], Bonavolontà P et al., 2019 [33], Schaper M et al., 2019 [32], Espinosa CA et al., 2018 [31], Infante-Cossio P et al., 2018 [18], Ogreden S et al., 2016 [30], Cristofaro MG et al., 2014 [29], Iro H et al., 2012 [28], Fukushima M et al., 2011 [27], Riad MA et al., 2011 [20], Uyar Y et al., 2011 [19], Guntinas O et al., 2004 [13], Piekarski J et al., 2004 [26], Gosh S et al., 2003 [25], Hancock BD et al., 1999 [24], Laskawi R et al., 1996 [23], Leverstein H et al., 1997 [22], McGurk M et al., 1996 [21].

Regarding facial nerve injury, the pooled analysis for transient facial nerve palsy using a random-effect model showed a rate of 5% in the ED group (*n* = 70/1208, 95% CI 3–8) vs. 26% in the SP group (*n* = 256/785, 95% CI 17–37), with substantial between-study heterogeneity in both groups (I^2^ = 64.3%, Q = 0.3004, *p* = 0.0028 vs. I^2^ = 85.6%, Q = 0.5851, *p* < 0.0001) (Figure 3A,B), while the pooled rate for permanent facial nerve palsy using a fixed-effect model was 3% in the ED group (*n* = 24/1316, 95% CI 2–4) vs. 4% in the SP group (*n* = 19/782, 95% CI 2–5), with a low between-study heterogeneity in both groups (I^2^ = 43.9%, Q = 0.4532, *p* = 0.0582 vs. I^2^ = 24.9%, Q = 0.2707, *p* = 0.2225) (Figure 3C,D). A statistically significant difference in the rate of transient facial nerve palsy was observed between the two groups (*p* < 0.05).

**Figure 4:**
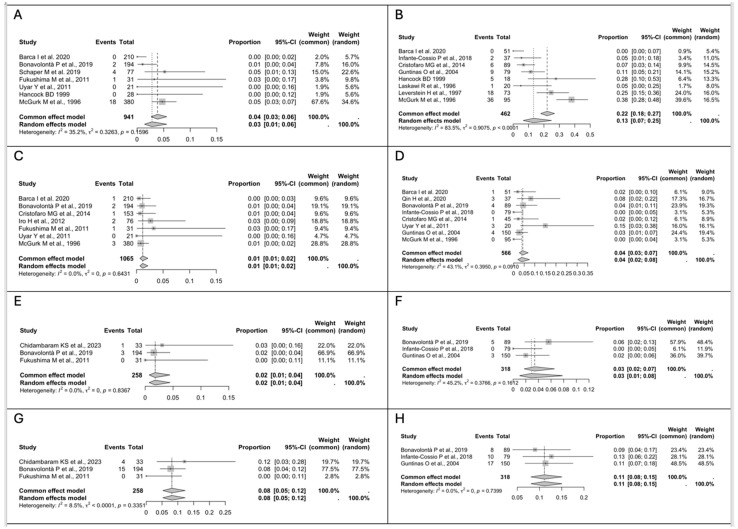
(**A**) Forest plot for Frey’s syndrome in the ED group. (**B**) Forest plot for Frey’s syndrome in the SP group. (**C**) Forest plot for salivary fistula in the ED group. (**D**) Forest plot for salivary fistula in the SP group. (**E**) Forest plot for seroma in the ED group. (**F**) Forest plot for seroma in the SP group. (**G**) Forest plot for hematoma in the ED group. (**H**) Forest plot for hematoma in the SP group. Abbreviations: CI, confidence interval. Data derived from Roh JL et al., 2024 [17], Chidambaram KS et al., 2023 [35], Barca I et al., 2020 [34], Qin H et al., 2020 [36], Bonavolontà P et al., 2019 [33], Schaper M et al., 2019 [32], Espinosa CA et al., 2018 [31], Infante-Cossio P et al., 2018 [18], Ogreden S et al., 2016 [30], Cristofaro MG et al., 2014 [29], Iro H et al., 2012 [28], Fukushima M et al., 2011 [27], Riad MA et al., 2011 [20], Uyar Y et al., 2011 [19], Guntinas O et al., 2004 [13], Piekarski J et al., 2004 [26], Gosh S et al., 2003 [25], Hancock BD et al., 1999 [24], Laskawi R et al., 1996 [23], Leverstein H et al., 1997 [22], McGurk M et al., 1996 [21].

As for the other post-operative complications, the pooled analysis for Frey’s syndrome using a fixed-effect model reported a 4% rate in the ED group (*n* = 25/941, 95% CI 3–6), with a low between-study heterogeneity (I^2^ = 35.2%, Q = 0.3263, *p* = 0.1596) (Figure 4A), while in the SP group, the pooled rate reported using a random-effect model was 13% (*n* = 77/462, 95% CI 7–25), with a considerable between-study heterogeneity (I^2^ = 83.5%, Q = 0.9075, *p* < 0.0001) (Figure 4B). The pooled rate for salivary fistula using a fixed-effect model was 1% in the ED group (*n* = 10/1065, 95% CI 1–2) vs. 4% in the SP group (*n* = 16/566, 95% CI 3–7), with a low between-study heterogeneity in both groups (I^2^ = 0%, Q = 0.0250, *p* = 0.5431 vs. I^2^ = 43.1%, Q = 0.3950, *p* = 0.0910) (Figure 4C,D). The incidence of Frey’s syndrome and salivary fistula differed significantly between the two groups, with the difference reaching statistical significance (*p* < 0.05). Seroma and hematoma were the other two post-operative complications analyzed. The pooled analysis for seroma using a fixed-effect model showed a 2% rate in the ED group (*n* = 4/258, 95% CI 1–4) vs. 3% rate in the SP group (*n* = 8/318, 95% CI 2–7), with a low between-study heterogeneity in both groups (I^2^ = 0%, Q = 0, *p* = 0.8367 vs. I^2^ = 45.2%, Q = 0.3766, *p* = 0.1612) (Figure 4E,F), while the pooled rate for hematoma using a fixed-effect model was 8% in the ED group (*n* = 19/258, 95% CI 5–12) vs. 11% in the SP group (*n* = 35/318, 95% CI 8–15), with a low between-study heterogeneity in both groups (I^2^ = 8.5%, Q < 0.0001, *p* = 0.3351 vs. I^2^ = 0%, Q = 0, *p* = 0.7399) (Figure 4G,H).

### 3.4. Risk of Bias Assessment

**Figure 5:**
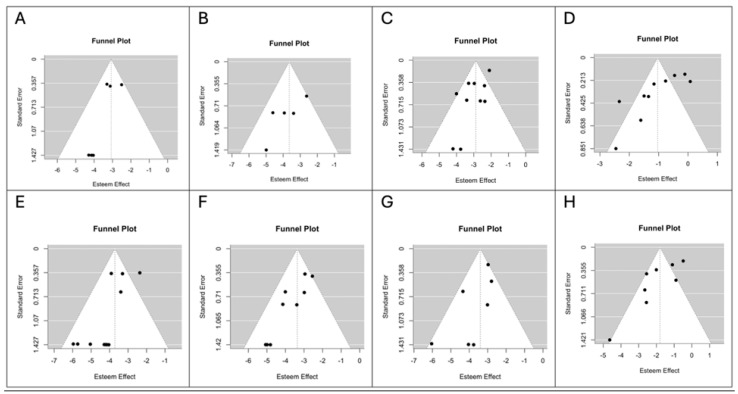
(**A**) Funnel plot for salivary fistula in the ED group. (**B**) Funnel plot for salivary fistula in the SP group. (**C**) Funnel plot for seroma in the ED group. (**D**) Funnel plot for seroma in the SP group. (**E**) Funnel plot for hematoma in the ED group. (**F**) Funnel plot for hematoma in the SP group. (**G**) Funnel plot for recurrence in the ED group. (**H**) Funnel plot for recurrence in the SP group. Abbreviations: CI, confidence interval.

**Figure 6:**
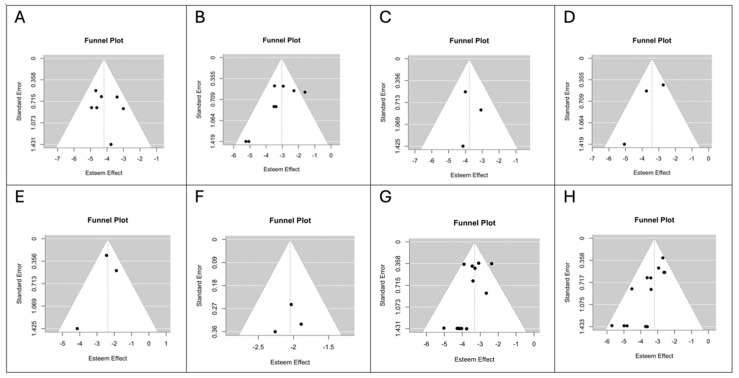
(**A**) Funnel plot for intraoperative capsular rupture in the ED group. (**B**) Funnel plot for intraoperative capsular rupture in the SP group. (**C**) Funnel plot for transient facial nerve palsy in the ED group. (**D**) Funnel plot for transient facial nerve palsy in the SP group. (**E**) Funnel plot for permanent facial nerve palsy in the ED group. (**F**) Funnel plot for permanent facial nerve palsy in the SP group. (**G**) Funnel plot for Frey’s syndrome in the ED group. (**H)** Funnel plot for Frey’s syndrome in the SP group.

The Newcastle–Ottawa Quality Assessment Scale scores of the individual studies are shown in Table 2. Funnel plots for each outcome are shown in Figure 5 and Figure 6. Visual inspection and the Egger’s linear regression test showed a symmetric distribution of the points in the funnel plots for intraoperative capsular rupture (t = −1.0374, *p* = 0.1694; Figure 5A), transient facial nerve palsy (t = −1.8362, *p* = 0.0663; Figure 5C), salivary fistula (t = 0.5193, *p* = 0.6036; Figure 6A) and recurrence (t = −1.4784, *p* = 0.1393; Figure 6G) in the ED group and for seroma (t = 0.2117, *p* = 0.8323 vs. t = −1.5117, *p* = 0.1306; Figure 6C,D; ED vs. SP) and hematoma (t = −0.9113, *p* = 0.3622 vs. t = −0.2489, *p* = 0.8035; Figure 6E,F; ED vs. SP) in both groups. A likely publication bias (*p* < 0.05) was detected instead for intraoperative capsular rupture (t = −1.9711, *p* = 0.0487; Figure 5B), transient facial nerve palsy (t = −3.0716, *p* = 0.0021; Figure 5D), salivary fistula (t = −2.0280, *p* = 0.0426; Figure 6B) and recurrence (t = −3.0508, *p* = 0.0023; Figure 6H) in the SP group. The same occurred for permanent facial nerve palsy (t = −2.3984, *p* = 0.0165 vs. t = −2.8350, *p* = 0.0046; Figure 5E,F; ED vs. SP) and Frey’s syndrome (t = −1.9995, *p* = 0.0456 vs. t = −2.8099, *p* = 0.0050; Figure 5G,H; ED vs. SP) in both groups.

## 4. Discussion

Pleomorphic adenoma is a slow-growing, well-defined, and typically mobile tumor that accounts for approximately 40–60% of benign salivary gland tumors and is found in the superficial lobe of the parotid gland in about 80% of cases [3,37]. The two primary objectives of parotid surgery for benign tumors are the complete removal of the lesion with adequate margins of healthy parotid tissue and the preservation of the facial nerve. Over the past century, various surgical techniques have been developed for treating benign parotid gland tumors [4,5,6,7]. In the early 20th century, intracapsular enucleation was commonly performed to minimize facial nerve damage. However, part or all of the (pseudo)capsule is retained in the operative field, leading to a high recurrence rate of 20% to 45% [8,9]. This resulted in the adoption of more radical surgical approaches. By the mid-20th century, SP emerged as the gold standard, significantly reducing recurrence rates to approximately 2% [10,11]. In the 1940s, Nicholson and Gleave introduced ED as an alternative surgical approach. This technique involves the removal of the tumor, either with a minimal cuff of normal parotid tissue or without any surrounding glandular tissue, without formally identifying the facial nerve [38], leading to comparable recurrence rates and fewer surgical complications [14,15].

Regarding the surgical technique, as early as 1940, Donovan and Conley [8] identified unfavorable key factors influencing the complete excision of parotid tumors: large tumor size and deep lobe involvement. However, Henriksson et al. [39] found no direct link between tumor size, location and recurrence rates. In their study, total parotidectomy was performed in all cases of deep-lobe PAs, yet tumor size itself was not associated with residual disease. The same finding occurred in Espinosa et al.’s series [31], where the mean tumor size in cases affected by a recurrence was 19.19 mm, and that in cases without recurrence was 21.84 mm (*p* = 0.273). Therefore, while size and location influence surgical technique, they do not inherently determine recurrence risk. Donovan and Conley [8] also identified tumors in the isthmus of the parotid gland and those close to the facial nerve as unfavorable factors. Witt et al. [7] further observed that dissecting a PA from the facial nerve frequently results in positive surgical margins. Studies analyzing resection margins consistently agree that close or positive margins significantly increase recurrence risk (*p* < 0.0001) [31]. This issue can be effectively managed with SP since the tumor is excised along with the entire superficial lobe, ensuring clear margins of more than 5 mm, whereas when ED is performed, only the area of the gland that involves the tumor is removed with a margin of 1.5 cm. However, the meta-analysis by Albergotti et al. [14] reported a similar risk for recurrence between the ED and SP techniques. This is in contrast with Foresta et al.’s meta-analyses [12], which found recurrence to be more common among patients with an indication for SP compared with patients who underwent ED (2.0 cases per 1000 person-years vs. 1.3 cases per 1000 person-years; pooled incidence rate).

Another issue to address in order to reduce the recurrence rate is the risk of intraoperative capsular rupture, which may lead to the dissemination of tumor cells and an increased likelihood of recurrence [17,34]. However, several studies found no significant differences in capsular rupture between the two techniques [29,34]. Regardless, the significance of accidental capsular rupture still remains unclear. Some studies suggest no statistically significant difference in recurrence rates between cases with and without intraoperative rupture (7–8% vs. 2.5–4%, respectively) [40,41], while others have identified a correlation [22,42].

Espinosa et al. [31] highlighted a significant relationship between adverse histopathologic characteristics and the severity of facial nerve dysfunction (*p* = 0.019). Specifically, patients with tumors exhibiting an incomplete capsule or pseudopodia were more likely to experience partial facial paresis. In contrast, the presence of satellite nodules was associated with a greater risk of complete facial paralysis. Furthermore, the severity of facial nerve impairment was notably influenced by positive surgical margins (*p* = 0.017) and intraoperative tumor rupture (*p* = 0.045). Bonavolontà et al. [33] observed a significant correlation between the surgical technique employed and the occurrence of facial nerve dysfunction (*p* < 0.001). Another main concern in the debate between ED and SP is indeed the potential for facial nerve branch dissection, which can result in permanent paralysis, as ED, compared to SP, does not require facial nerve identification and dissection. Witt et al. [42] highlighted that the amount of parotid tissue resected is directly proportional to the rate of Frey’s syndrome and transient nerve dysfunction, reporting a two-fold increase in transient facial nerve dysfunction in SP compared to ED and a 1.8-fold higher rate of permanent facial nerve dysfunction in ED than in SP. This complication does not always stem from nerve branch neurotmesis; rather, it can result from surgical manipulation, leading to temporary nerve injury, with the marginal mandibular branch as the most frequently affected [17]. The risk of such injury is closely related to the duration of nerve exposure during the procedure [26]. In ED, the facial nerve remains unexposed unless it is in direct contact with the tumor. When contact occurs, only a small portion of the nerve branches is handled, minimizing the risk of damage. These findings suggest that ED may be a more favorable technique for preserving facial nerve function.

Other relevant post-operative complications that must be considered when performing parotid gland surgery are Frey’s syndrome, salivary fistula and local complications like seroma and hematoma. ED is associated with a lower incidence of post-operative complications, primarily due to its minimally invasive approach, which limits disruption of the parotid parenchyma and preserves parasympathetic innervation. As mentioned above, Witt et al. [42] emphasized that the extent of parotid tissue resection is directly correlated with the incidence of Frey’s syndrome, with a ten times higher rate following SP compared to ED.

In this context, a critical review of the literature was conducted covering the years from 1950 to 2025. We focused our analyses on PA, reporting on the main concerns that over the years contributed to the debate between SP and ED as the favorable surgical approach for PA.

To the best of our knowledge, the most recent meta-analysis and review on the surgical treatment of PAs dates to 2014 [12]. As opposed to Foresta et al.’s meta-analysis [12], we observed a similar risk for recurrence after ED and SP (3% vs. 4%, respectively) with low between-study heterogeneity in both groups. Moreover, the risk of intraoperative capsular rupture was similar in the two groups (4% vs. 2%), though this might not be related to the risk of relapse as reported in the literature. In line with Foresta et al. [12], a higher rate for facial nerve paralysis and Frey’s syndrome was found in the SP group (5% vs. 26% and 4% vs. 13%, respectively; *p* < 0.05); however, in our series, the rate of permanent facial nerve palsy was similar in the two groups (3% vs. 4%), probably due to the facial nerve branch dissection during SP that leads to temporary nerve injury. Additionally, salivary fistula (*p* < 0.05), seroma and hematoma results were lower after ED given the minimally invasive approach that minimizes parotid tissue resection while preserving the parasympathetic nerve supply, thereby reducing the risk of functional impairment and post-operative complications.

Our literature review has not identified any randomized clinical trials directly comparing ED and SP, preventing a direct evaluation of the two techniques. In reviewing the literature, we observed variability in study design, differences in sample sizes, and inconsistent follow-up durations that may introduce biases. Furthermore, some outcomes may be affected by potential publication bias as reported above.

The primary source of bias identified was heterogeneity across studies, including differences in inclusion criteria, reinterventions, disease stage and type, and tumor size, all of which limit the validity of the meta-analysis. Moreover, the presence of satellite nodules—an important factor influencing recurrence risk in ED—was inconsistently reported and could not be systematically assessed.

Another significant limitation is the lack of detailed information on surgeon experience, training and practice setting. While some studies mentioned general criteria for surgical selection, such as tumor size and location, none clarified whether the choice between ED and SP was based on institutional policies, surgeon preference or predefined protocols. This lack of standardization in surgical decision-making introduces potential selection bias.

We recommend that future studies standardize reporting of tumor characteristics (e.g., size, location, histologic subtype and features), surgeon-related variables and institutional approaches to surgical decision-making in order to improve comparability and clinical applicability of findings.

## 5. Conclusions

This meta-analysis concluded that ED may be the treatment of choice for pleomorphic adenomas of the parotid gland that are up to 3 cm in size, mobile and located in the superficial lobe of the parotid gland. In particular, our findings showed the following:

(1) The risk of recurrence is similar in patients treated with ED and SP.
(2) The transient facial nerve palsy rate is lower after ED, while the permanent facial nerve palsy rate is similar for ED and SP.
(3) Post-operative complications, especially Frey’s syndrome, are more common after SP.

## Figures and Tables

**Table 1:** Characteristics of included studies; * = Median.

Title, Year	Study Design	No. Patients	Mean Age	Mean Follow Up (Range) (ED/SP)	Mean Tumor Size (Range) (ED/SP)	Localization	ED	SP	Capsular Rupture (ED/SP)	Transient Facial Narve Palsy (ED/SP)	Permanent Facial Nerve Palsy (ED/SP)	Frey’s Syndrome (ED/SP)	Salivary Fistula (ED/SP)	Sialocele (ED/SP)	Seroma (ED/SP)	Hematoma (ED/SP)	Recurrence (ED/SP)
Roh JL et al., 2024 [17]	Prospective	36	54	44 mo (24–60)	2.8 cm	Deep or superficial parotid lobe	36		0	2	0			3		4	0
Chidambaram KS et al., 2023 [35]	Retrospective	33	32.75	(2–43) mo	(2–4) cm	Mainly the tail of the superficial lobe, with some having minimal superior or deep lobe extension	33			0	0				1		0
Barca I et al., 2020 [34]	Retrospective	261	47	65 mo	2.5/2.8 cm	Superficial parotid lobe with a diameter of 3.0 (0.5) cm	210	51	7/3	7/9	0/2	0/0	1/1				6/3
Qin H et al., 2020 [36]	Retrospective	75		24 mo		Superficial parotid lobe		37				2	3				
Bonavolontà P et al., 2019 [33]	Retrospective	297	52	43 (25–168) mo *		ED: superficial parotid lobe SP: deep parotid lobe	194	89		3/21	0/6	2/6	2/4		3/5	15/8	8/4
Schaper M et al., 2019 [32]	Retrospective	205	48	7.9 y		Deep or superficial parotid lobe	77			2	0	4					2
Espinosa CA et al., 2018 [31]	Retrospective	198	46.18	56.7 (1–437) mo	2.1 cm	180: superficial parotid lobe 18: deep parotid lobe		165		78							10
Infante-Cossio P et al., 2018 [18]	Prospective	79	48	30.65 (12–45) mo	2.44 (1–6) cm	Superficial parotid lobe		79		41	0	9	0	0	0	10	
Ogreden S et al., 2016 [30]	Retrospective	50		5 y		Superficial parotid lobe		18		1		5					0
Cristofaro MG et al., 2014 [29]	Retrospective	198	50.97	61.02/66.4 mo	3.0/2.5 cm	Superficial parotid lobe	153	45	6/1	7/9	0/1		1/1				5/1
Iro H et al., 2012 [28]	Retrospective	76	51.35	7.38 (5.05–10.52) y		Superficial parotid lobe	76			6	2		2				0
Fukushima M et al., 2011 [27]	Retrospective	31	43	61 (18–125) mo	2.8 cm	Deep or superficial parotid lobe	31		0		0	1	1	0	0	0	0
Riad MA et al., 2011 [20]	Prospective	164	49.5	56.4 (34–93) mo		141: superficial parotid lobe 19: deep parotid lobe 22: parapharyngeal space		77	1								2
Uyar Y et a l., 2011 [19]	Prospective	41	48.8	194 (117–264) mo		Superficial parotid lobe	21	20		0/3		0/1	0/3				0/0
Guntinas O et al., 2004 [13]	Retrospective	295	253	8 y		Deep or superficial parotid lobe		150	1	58	7		4		3	17	0
Piekarski J et al., 2004 [26]	Retrospective	98		34.3 mo	3.6 (1.5–10) mo	N/A	98		7		8						8
Gosh S et al., 2003 [25]	Retrospective	83	49.4	10.6 mo (0–10.5 y)		N/A	22	49									1/3
Hancock BD et al., 1999 [24]	Retrospective	101	43/46	10.3 (3–21)/8.3 (3–22) y		N/A	28	73	0/0	2/6	0/0	0/18					0/0
Laskawi R et al., 1996 [23]	Retrospective	475		63 mo		N/A		139			2	20					1
Leverstein H et al., 1997 [22]	Retrospective	245		95 mo		N/A		61			0	8					0
McGurk M et al., 1996 [21]	Retrospective	475	47	12.5 (1–34) y		N/A	380	95		41/30	7/1	18/36	3/0				7/2

**Table 2:** Newcastle–Ottawa Quality Assessment Scale scores of included studies. xxx = high quality; xx = moderate quality; x = low quality.

Study	Selection	Comparison	Outcome
Roh JL et al., 2024 [17]	xxx	x	xxx
Chidambaram KS et al., 2023 [35]	xxx	x	xxx
Barca I et al., 2020 [34]	xx	xx	xx
Qin H et al., 2020 [36]	xxx	x	xx
Bonavolontà P et al., 2019 [33]	xxx	xx	xxx
Schaper M et al., 2019 [32]	xxx	x	xxx
Espinosa CA et al., 2018 [31]	xxx	xx	xxx
Infante-Cossio P et al., 2018 [18]	xxx	x	xx
Ogreden S et al., 2016 [30]	xxx	x	xx
Cristofaro MG et al., 2014 [29]	xxx	x	xxx
Iro H et al., 2012 [28]	xxx	x	xxx
Fukushima M et al., 2011 [27]	xxx	x	xxx
Riad MA et al., 2011 [20]	xxx	x	xx
Uyar Y et al., 2011 [19]	xxx	xx	xx
Guntinas O et al., 2004 [13]	xxx	x	xxx
Piekarski J et al., 2004 [26]	xxx	x	xxx
Gosh S et al., 2003 [25]	xxx	x	xxx
Hancock BD et al., 1999 [24]	xxx	x	xx
Laskawi R et al., 1996 [23]	xxx	x	xx
Leverstein H et al., 1997 [22]	xxx	x	xxx
McGurk M et al., 1996 [21]	xxx	x	xxx

## Data Availability

The data that support the findings of this study are available from the corresponding author upon reasonable request.

## Supplementary Materials

The following supporting information can be downloaded at: https://www.mdpi.com/article/10.3390/medsci13030104/s1, Table S1: GRADE (Grading of Recommendations Assessment, Development and Evaluation) of included studies.
