# Supplementary material for: Pleomorphic Adenoma: Extracapsular Dissection vs. Superficial Parotidectomy—An Updated Systematic Review and Meta-Analysis
Source: Med Sci (Basel). 2025 Jul 31;13(3):104. doi: 10.3390/medsci13030104 (PMC12372145; doi:10.3390/medsci13030104)
Supplement: Supplementary file 1 [file medsci-13-00104-s001.zip › medsci-3739647-supplementary.pdf]

Table S1. GRADE (Grading of Recommendations Assessment, Development and Evaluation) of included studies.

Outcome	Type of evidence	Risk of bias	Inconsistency	Imprecision	Indirectness	Publication bias	Overall certainty
Recurrence	Observational studies	**Serious** (retrospective design, selection bias)	**Not serious** (low heterogeneity: I² = 4.8–13%)	**Not serious** (large n, narrow CI)	**Not serious**	**Suspected** (Egger's test p = 0.0023 for SP)	●●○○ Low
Intraoperative capsular rupture	Observational studies	**Serious**	**Not serious** (I² = 0–25%)	**Not serious**	**Not serious**	**Suspected** (p = 0.0487 for SP)	●●○○ Low
Transient facial nerve palsy	Observational studies	**Serious**	**Serious** (high heterogeneity: I² = 64–85%)	**Not serious**	**Not serious**	**Likely** (p = 0.0021 for SP)	●○○○ Very Low
Permanent facial nerve palsy	Observational studies	**Serious**	**Not serious** (I² = 25–44%)	**Not serious**	**Not serious**	**Likely** (p = 0.0046–0.0165 both groups)	●●○○ Low
Frey's syndrome	Observational studies	**Serious**	**Serious** (SP: I² = 83.5%)	**Not serious**	**Not serious**	**Likely** (p = 0.0050 for SP)	●○○○ Very Low
Salivary fistula	Observational studies	**Serious**	**Not serious** (I² = 0–43%)	**Not serious**	**Not serious**	**Suspected** (p = 0.0426 for SP)	●●○○ Low
Seroma	Observational studies	**Serious**	**Not serious** (I² = 0–45%)	**Serious** (few events, wide CI)	**Not serious**	**Unlikely** (Egger's test p > 0.13)	●●○○ Low
Hematoma	Observational studies	**Serious**	**Not serious** (I² = 0–8.5%)	**Serious** (low number of events)	**Not serious**	**Unlikely** (p > 0.36 both groups)	●●○○ Low
